# Breast Cancer Screening: Knowledge, Attitudes, and Practices among Female University Students in The Gambia

**DOI:** 10.1155/2023/9239431

**Published:** 2023-11-30

**Authors:** Bakary Kinteh, Sambou L. S. Kinteh, Amienata Jammeh, Ebrima Touray, Amadou Barrow

**Affiliations:** ^1^School of Public Health, Gambia College, Brikama Campus, West Coast Region, Gambia; ^2^Department of Public & Environmental Health, School of Medicine & Allied Health Sciences, University of The Gambia, Kanifing, Gambia

## Abstract

**Background:**

Breast cancer is the second most prevalent form of cancer in The Gambia, with an incidence rate of approximately 15% and a mortality rate exceeding 50% in 2020. The all-age prevalence stands at 11.25 per 100,000 population. In light of this, we conducted a study to assess the knowledge, attitude, and practice of breast cancer screening among female university students.

**Method:**

We conducted an institutional cross-sectional study involving 361 randomly sampled female university students. Data collection was done using a pretested, self-administered questionnaire. We utilized descriptive statistics to describe the prevalence and burden of breast cancer screening among the participants.

**Results:**

Our study revealed good knowledge regarding breast cancer screening among female university students, yet 82.8% had a negative attitude about the disease. More than three-quarters (76.6%) of the respondents had never practiced any form of breast cancer screening. Notably, there was a significant association between knowledge of breast cancer screening and attitude (*p* = 0.027), and factors such as level of study (*p* = 0.041), ethnicity (*p* = 0.026), parity (*p* = 0.018), and faculty of study (*p* = <0.001) influenced the participants' knowledge.

**Conclusion:**

It is crucial to implement comprehensive awareness campaigns to address the negative attitude and poor screening practices among female university students regarding breast cancer. Additionally, providing free and widespread breast cancer screening services to students should be considered as a means to combat this disease.

## 1. Introduction

Breast cancer is a disease in which cells in the breast grow out of control [1], and it is the most prevalent cancer among women worldwide, affecting both developed and developing countries [2]. In 2017, there were approximately 2 million cases of breast cancer globally, with a mortality rate exceeding 50% [3]. It is estimated that up to 13% of women worldwide will experience breast cancer in their lifetime [4]. Although there are marginal variations in breast cancer prevalence between developed and developing countries, it remains a significant health issue globally.

The sub-Saharan Africa (SSA) region is facing an increasing burden of noncommunicable diseases, including cancer, which is emerging as a public health concern [3, 5]. In The Gambia, breast cancer is the second most prevalent cancer among women, and its incidence is notably rising. In 2020, the incidence rate of breast cancer was 15%, with a mortality rate exceeding 50% among diagnosed patients. The five-year all-age prevalence of breast cancer was 11.25 per 100,000 population [6].

The World Health Organization (WHO) has developed a global breast cancer initiative framework with the goal of saving nearly three million lives from breast cancer by 2040. This framework focuses on health promotion for early detection, timely diagnosis, and proper management of breast cancer cases [7]. Breast self-examination is a fundamental tool in empowering women to detect any abnormalities in their breasts [8]. The survival rate for women with breast cancer depends heavily on early detection [9].

In SSA, the diagnosis of breast cancer often occurs at an advanced stage, leading to poor prognosis. The survival rate for breast cancer patients in low- and middle-income countries is less than 0.5% [10]. Several factors contribute to the delayed diagnosis of breast cancer in SSA, including limited access to modern diagnostic facilities, inadequate knowledge about breast cancer, misconceptions, and sociocultural factors [11, 12]. In The Gambia, access to breast cancer screening services is a significant public health concern, with only 52 out of 102 health facilities providing clinical breast examination and a limited number of facilities offering pathological and surgical management of breast cancer. Additionally, the distance to access these facilities ranges from 10 to 45 kilometers [5].

Unfortunately, breast cancer has a significant impact on the health and well-being of women in the country, with unsatisfactory outcomes in terms of diagnosis, treatment, and prognosis. However, early detection through breast cancer screening is crucial and offers the best chance for effective management and prevention. The deficiency of breast cancer screening facilities is a national health problem that hinders the achievement of universal health coverage for women's health. Screening facilities are often centralized, and the cost of services is not affordable for many economically disadvantaged women [5]. Additionally, the incidence of breast cancer in The Gambia is increasing [6], and effective strategic interventions are needed to curtail the disease based on tangible research findings.

Thus, this study is aimed at assessing the knowledge, attitude, and practices of breast cancer screening among female students at the University of The Gambia. By conducting this study, we hope to identify gaps in the knowledge, attitude, and practices related to breast cancer prevention among university students. The findings from this study can inform policy decisions and design and execute feasible interventions to prevent breast cancer and contribute to awareness creation on breast cancer prevention among the population.

## 2. Methodology

### 2.1. Study Design and Setting

The study was conducted at the University of The Gambia using an institutional cross-sectional design to examine the knowledge, attitude, and practices of female students regarding breast cancer screening. The University of The Gambia is the country's sole state-owned university, established by an act of Parliament in 1999 [13]. It comprises nine schools or faculties and has an estimated student population of seven thousand [13]. The university's campuses are situated in Banjul, Kanifing, Brikama, Faraba, and Farafenni [14].

### 2.2. Study Population

The study was carried out among female students enrolled in courses or programs at the University of The Gambia. Only female students within the age range of 18 to 49 were included in the study, provided they gave their consent. Female students who had already completed their coursework at the university were excluded from the study.

### 2.3. Sample Size Determination and Sampling

A sample size of 361 students was determined using the Kish Leslie formula (1964): *n* = *z*^2^*pq*/*e*^2^, where *p* is the proportion of knowledge on breast cancer among female students (31%), *e* is the desired level of precision (5%), and *z* = 1.95 at CI 95% [15]. The calculation also included a 10% nonresponse rate.

This study utilized a multistage sampling technique. Firstly, all nine faculties were purposively included as part of the sample. Secondly, a proportionate sampling strategy was employed to recruit study participants from each faculty. Thirdly, participants were selected based on their level of study using another proportionate sampling method. Finally, a simple random sampling approach was employed to recruit 361 female students who would participate in the study.

### 2.4. Data Collection

The researchers utilized closed-ended structured questionnaires that were adapted from previous studies conducted among female university students [2, 8, 15–19]. These questionnaires were organized into four sections: sociodemographic information, knowledge about breast cancer, attitude towards breast cancer, and breast cancer screening practices. The sociodemographic section collected information such as the respondent's age, faculty of study, level of study, marital status, religion, ethnicity, parity, and age at menarche.

The knowledge section consisted of fifteen questions related to breast cancer, screening methods, risk factors, sources of information, and signs and symptoms. The knowledge questions were dichotomized as (yes = 1, no = 0) which were used to calculate the composite knowledge score. A composite score of ≥50% is classified as good knowledge while ≤50% is scored poor knowledge. Attitude was assessed using a Likert scale of 5 point based on six questions. The questions were asked evenly 3 positive and negative questions. The composite attitude was scored as good attitude (≥50%) and poor attitude (≤50%). The section on breast cancer screening practices included six questions about the performance of breast cancer screening, the method used, reasons for screening or not screening, who should practice screening, and the frequency of screening. The composite practice score was also calculated using the six questions and was classified as good practice (≥50%) and poor practice (≤50%).

Data collection was carried out using self-administered questionnaires distributed to the students, who were then requested to complete and return them to the researchers. The data collection period took place from September to November 2022. To ensure confidentiality, no personal identifiers such as names or mobile numbers were linked to the respondents. Additionally, participants were provided with envelopes to seal the completed questionnaires before submission. Prior to the study, the questionnaire was pilot-tested among female students at Gambia College School of Public Health to ensure consistency and reliability.

### 2.5. Data Analysis

The data was analyzed using IBM Statistical Package for Social Science (SPSS) version 26.0. The continuous variables were summarized into mean and standard deviation while the categorical variables were summarized into frequency and percentage.

### 2.6. Ethical Considerations

The ethical clearance was obtained from Gambia College Research and Consultancy unit, the Directorate of Research at the University of The Gambia, and participants signed an informed consent form to participate in the study.

## 3. Results

In this study, a total of 361 female students from the University of The Gambia participated, resulting in a response rate of 98.3% (*n* = 355). The findings from Table 1 revealed several key characteristics of the respondents. The mean age of the participants was 22.9 ± 3.857 years, with the age range of 18-24 years representing the majority of respondents at 78%. Among the participants, more than 31% identified themselves as Mandinka ethnicity, while 88.5% practiced Islam as their religion. In terms of academic affiliation, the largest proportion (28.5%) of respondents were recruited from the School of Arts and Sciences, and nearly 45% were classified as sophomores. Furthermore, approximately 80% of the participants were single, 89% were nullipara (having no children), and the majority (61.7%) experienced their menarche at an average age of 13.54 ± 1.38 years.

### 3.1. Knowledge on Breast Cancer

According to the findings presented in Table 2, all of the respondents (100%) reported being aware of breast cancer. The majority of them (more than 62%) acquired this knowledge through mass media. It is noteworthy that over half of the participants demonstrated knowledge of both clinical and breast self-examination methods. However, a significant proportion, 78.6% and 66.5%, respectively, lacked awareness of the correct positioning and appropriate timing for breast self-examination. Additionally, nearly 82% of the respondents recognized the benefits of breast screening in detecting breast cancer at an early stage, while 83.1% acknowledged that early detection could improve the chances of survival. Several commonly reported risk factors for breast cancer included being a woman, having a family history of breast cancer, leading a sedentary lifestyle, and engaging in tobacco or alcohol consumption. The composite knowledge score of the study indicated a good level of knowledge, reaching 54.4%.

**Figure 1:**
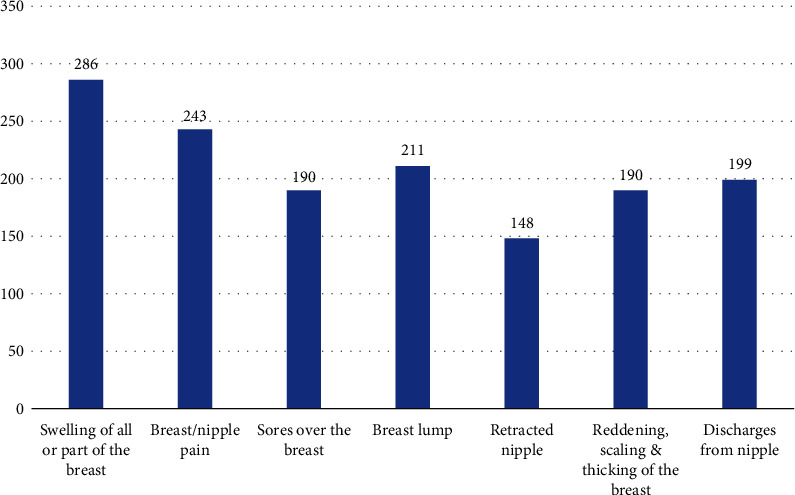
Respondents' knowledge on signs and symptoms of breast cancer.

A majority of the respondents demonstrated awareness of the common signs and symptoms of breast cancer. Specifically, 80.6% of the participants were able to identify swelling of all or part of the breast as a symptom. Additionally, 68.5% recognized breast/nipple pain, and 59.4% were aware of the presence of breast lumps as potential indicators of breast cancer as in Figure 1.

### 3.2. Attitude on Breast Cancer Screening

The findings shown in Table 3 show the attitude of the respondents towards breast cancer screening which was evaluated through six questions. The findings indicate that a significant portion of the respondents held positive attitudes. Specifically, 60% strongly agreed that all females should undergo breast cancer screening, emphasizing its importance. Furthermore, 68.7% strongly agreed that breast cancer screening plays a crucial role in detecting breast abnormalities, highlighting its significance in early detection. Additionally, 44.8% strongly agreed that early detection of breast cancer can help prevent further complications, emphasizing the benefits of timely screening. In contrast, a notable percentage of respondents expressed negative attitudes. Specifically, 43.4% strongly disagreed with the notion that breast cancer survival depends on early detection, suggesting a lack of belief in its impact. Similarly, 50.1% strongly disagreed that breast self-examination aids in the detection of breast cancer, indicating skepticism towards its effectiveness. Additionally, 43.1% strongly disagreed that women with a family history of breast cancer should be concerned about the disease, suggesting a dismissive attitude towards the relevance of family history. Overall, the study revealed a negative attitude score of 82.8%, indicating that a significant portion of the respondents had unfavorable attitudes towards breast cancer screening.

### 3.3. Practice of Breast Cancer Screening

From Table 4, only 23.4% of the respondents in the study reported having ever practiced any form of breast cancer screening. Specifically, 14.4% had undergone clinical breast examination, with 20% of those individuals doing so based on the recommendation of a healthcare professional. When asked about the reasons for not performing breast cancer screening, 7.4% cited the absence of any noticeable signs or symptoms, while 62.0% believed that women of reproductive age should undergo breast cancer screening. Furthermore, over 20% of the respondents stated that breast cancer screening should be conducted on a monthly basis. Overall, the study revealed a total practice score of 82.3%, indicating a poor level of adherence to breast cancer screening practices among the respondents.

## 4. Discussion

Good knowledge, positive attitude, and practices are a prerequisite in fight against and prevention of breast cancer. In our study, we found that all respondents (100%) had heard about breast cancer, and over 50% of them demonstrated good knowledge about breast cancer screening. This finding aligns with a study conducted among university students in Bahauddin Zakariya University, Pakistan, where 100% of the students were aware of breast cancer [20]. Media emerged as the primary source of information on breast cancer for the majority of our respondents (62.8%), followed by health professionals (33%). This underscores the critical role of media in raising health awareness. It is worth noting that our study coincided with Breast Cancer Awareness Month in October, which was also celebrated by the University Students' Union.

Furthermore, our respondents displayed good knowledge of breast cancer screening methods, with over half of them being aware of both clinical breast examination and breast self-examination. However, a significant proportion (nearly 70%) lacked knowledge about the appropriate timing for breast self-examination. On the other hand, the majority of our respondents demonstrated good knowledge of breast cancer signs. This suggests that continuous sensitization and awareness campaigns have played a role in increasing knowledge about the disease. Given that reproductive health issues are of universal concern, it is crucial to equip university students with the necessary knowledge about their reproductive health and rights. In conclusion, battling breast cancer among women begins with knowledge and awareness of its signs and risk factors.

Regarding knowledge of risk factors, our study used 12 items to assess respondents' understanding. Interestingly, many of them correctly identified obesity (35.2%), family history of breast cancer (57.5%), being a woman (60.3%), tobacco/alcohol consumption (47.3%), and a sedentary lifestyle (40.8%) as risk factors for breast cancer. These findings are lower compared to breast cancer awareness studies among university students conducted in other regions [2, 16, 21, 22]. However, our respondents demonstrated limited knowledge about oral contraceptives as a risk factor, which may be attributed to the low contraceptive usage rate in the country, as less than 20% of reproductive-age women use contraceptives [23]. Moreover, there is a knowledge gap regarding risk factors such as early onset of menses below 12 years and late menopause. These findings are lower compared to studies conducted in Jordan, Kampala, and Makkah [2, 19, 22].

A significant number of our survey participants demonstrated knowledge of the correct signs and symptoms of breast cancer, such as breast swelling, breast/nipple pain, breast lumps, and nipple discharge. These findings align with previous studies conducted in Uganda, Malaysia, and Jordan, where respondents correctly identified these signs and symptoms of breast cancer [2, 16, 21]. However, a considerable proportion of our respondents lacked awareness of retracted nipples as an important sign of breast cancer, indicating a knowledge gap in recognizing signs and symptoms.

Our study revealed that more than 80% of respondents held a negative attitude towards breast cancer. This negative attitude towards breast cancer screening may stem from fears about potential findings and influences from sociocultural and religious factors. On the other hand, respondents displayed a positive attitude towards seeking healthcare and preventing breast cancer. These positive attitudes might be influenced by awareness of breast cancer, knowledge of risk factors, and awareness of signs and symptoms. It is evident that continuous efforts are necessary to enhance awareness among females regarding breast cancer screening and improve their attitudes towards it.

Although our respondents demonstrate good knowledge about breast cancer screening, more than two-thirds (76.6%) of them reported never having practiced any screening method. This finding is consistent with a high composite poor practice score of 82.3%, which exceeds the rates found in studies conducted in Cameroon, Jordan, Uganda, Malaysia, and the United Arab Emirates [2, 16, 18, 21, 22, 24–27]. The breast cancer screening methods that have been practiced include breast self-examination (BSE) by 9.0% of respondents and clinical breast examination (CBE) by 14.4%. However, none of our respondents have undergone mammography. This finding supports the fact that CBE is the most commonly performed breast cancer screening method in the country, available in 52 out of 102 health facilities [5].

Furthermore, our claim that healthcare-seeking behavior reflects a positive attitude among respondents is supported by the fact that 20% of those who performed breast cancer screening were advised by a healthcare professional. However, the main reasons cited for not practicing breast cancer screening were lack of knowledge (32.4%), fear of discovering a mass (22.1%), forgetfulness (16.5%), and lack of time (12.9%). Improving the practice of breast cancer screening can be achieved through increased awareness campaigns, the provision of breast examination services in more health facilities, addressing negative attitudes towards screening among females, and implementing mass screening initiatives for breast cancer in the population. Our study also revealed a significant association between knowledge of breast cancer screening and attitudes towards breast cancer (*p* value = 0.027). Good knowledge of breast cancer screening is influenced by the respondent's level of education (*p* value 0.041), ethnicity (*p* value = 0.026), parity (*p* value = 0.018), and faculty of study (*p* value < 0.001).

### 4.1. Limitations of the Study

The findings of this study are limited in their generalizability to the overall female population of the country due to the exclusive focus on female university students as participants. Additionally, potential information bias may have resulted from the self-reporting of data, and respondents were not categorized based on their medical or nonmedical backgrounds. Furthermore, the study did not assess the participants' ability to demonstrate the proper technique for performing breast self-examination (BSE), which could have impacted their ability to correctly practice BSE.

## 5. Conclusion

All of our study participants knew about breast cancer, as they were familiar with the disease. The composite knowledge score among female university students regarding breast cancer was 54.4%. However, despite this good knowledge, 8 out of 10 respondents had a negative attitude towards breast cancer screening. It is crucial to transform this knowledge into a positive attitude to effectively combat breast cancer among females. Additionally, the study revealed poor practices of breast cancer screening. More than three-quarters of the respondents had never practiced any form of screening. Among those who had engaged in screening, the methods used were breast self-examination (9.0%) and clinical breast examination (14.4%), with no respondents having undergone mammography. Those who had performed breast cancer screening had been advised to do so by a healthcare professional.

In conclusion, it is essential to convert knowledge about breast cancer screening into positive attitudes and practices to make significant progress in the fight against breast cancer among females. Additionally, the findings could be used for awareness creation among university students, free breast cancer screening for students, and a curriculum on reproductive health issues and rights will complement the efforts of breast cancer prevention. Further studies focusing on breast self-examination are needed.

## Figures and Tables

**Table 1:** Sociodemographic characteristics of respondents.

Variables	*n*	%
Age of participants (in years)		
18-24	227	78.0
25-31	66	18.6
32-38	7	2.0
39-45	5	1.4
Ethnicity		
Mandinka	112	31.5
Fula	63	17.7
Wolof	74	20.4
Serahuli	14	3.9
Manjago	31	8.7
Serer	11	3.1
Jola	34	9.6
Others	16	7.5
Religion		
Islam	324	88.5
Christianity	41	11.5
Faculty/school of study		
Agriculture and Environmental Sciences	16	4.5
Arts and Sciences	101	28.5
Business and Public Administration	82	23.1
Education	15	4.2
Engineering and Architecture	9	2.5
Information Technology and Communication	13	3.7
Journalism and Digital Media	21	5.9
Law	22	6.2
Medicine and Allied Health Sciences	76	21.4
Year of study		
Freshman	93	26.2
Sophomore	159	44.8
Junior	67	18.9
Senior	36	10.1
Marital status		
Single	280	78.9
Married	75	21.1
Parity		
Nullipara	316	89.0
Primipara	31	8.7
Multipara	8	2.3
Age at menarche (in years)		
10 to 12	97	27.3
13-15	219	61.7
16 and above	39	11.0

**Table 2:** Knowledge of respondents on breast cancer.

Variable	*n*	%
Heard about breast cancer		
Yes	355	100%
Source of information		
Mass media	223	62.8
Health professional	117	33
School	86	24.2
Friends	52	14.6
Family member	30	8.5
Knew breast examination methods^∗^		
Clinical breast examination	208	58.4
Mammography	121	34.1
Breast self-examination	195	54.9
Knew positions of breast self-examination (BSE)		
Standing	30	9.3
Lying	28	7.9
In the shower	18	5.1
Do not know	279	78.6
knew the suitable period to perform BSE		
Before menstruation	12	3.4
During menstruation	29	8.2
5th to 7th day of menstrual cycle	27	7.6
Anytime	51	14.4
Do not know	236	66.5
Knew breast screening is helpful for early detection of breast cancer		
Yes	290	81.7
No	24	6.8
Do not know	41	11.5
Knew early detection of breast cancer can improve survival		
Yes	295	83.1
No	7	2
Do not know	53	14.9
Knew risk factors of breast cancer^∗^		
Obesity/overweight	125	35.2
Old age	80	22.5
Family history of breast cancer	204	57.5
Birth of first child after the age of 30 years	55	15.5
Early onset of menses (before age 12)	55	15.5
Late menopause (after 55 years)	40	11.3
Late initiation of breastfeeding	138	38.9
Being a woman	214	60.3
Cigarette smoking/alcohol consumption	168	47.3
Sedentary lifestyle	145	40.8
Use of oral contraceptive	80	22.5
Composite knowledge score		
Low knowledge	162	45.6
Good knowledge	193	54.4

^∗^Multiple responses.

**Table 3:** Attitude of respondents on breast cancer screening.

Attitude item	*N*	Mean	Std. dev	SA (*n*, %)	A (n, %)	U (*n*, %)	D (*n*, %)	SD (*n*, %)
All females need to perform breast cancer screening	355	4.52	0.648	213 (60.0)	118 (33.2)	21 (5.9)	3 (0.8)	0 (0.0)
Breast cancer screening is important to detect breast cancer early	355	4.60	0.644	244 (68.7)	82 (23.1)	28 (7.9)	1 (0.3)	0 (0.0)
Early detection of breast helps prevent further complications	355	4.16	0.967	159 (44.8)	126 (35.5)	43 (12.1)	21 (5.9)	6 (1.7)
Breast cancer survival does not depend on early detection	355	4.07	1.09	21 (5.9)	10 (2.8)	50 (14.1)	120 (33.8)	154 (43.4)
Breast self-examination does not help in detecting breast cancer	355	4.52	0.621	1 (0.3)	6 (1.7)	70 (19.7)	100 (28.2)	178 (50.1)
A woman with family history of breast cancer should not worry about breast cancer	355	3.71	1.40	30 (8.5)	50 (14.1)	40 (11.3)	82 (23.1)	153 (43.1)

SA = strongly agreed; A = agreed; U = undecided; D = disagreed; SD = strongly disagreed.

**Table 4:** Practices of breast cancer screening.

Variables	*n*	%
Have you ever practiced any breast cancer screening?		
Yes	83	23.4
No	272	76.6
Which breast screening method?		
Breast self-examination	32	9.0
Clinical breast examination	51	14.4
Mammography	0	0.0
Reasons for breast screening		
Advice from a health worker	71	20.0
Based on medical condition	4	1.1
Noticed a breast lump	8	2.3
Reasons for not breast screening		
Lack of knowledge	88	32.4
Did not have any sign	20	7.4
Forgetfulness	45	16.5
Lack of time	35	12.9
Fear of the finding a mass	60	22.1
Cost	24	8.8
Who should practice breast cancer screening		
Old women	12	3.4
Adolescents	110	31.0
Pregnant women	13	3.7
Women in their reproductive age	220	62.0
How often should breast screening be performed		
Daily	9	2.5
Weekly	7	2.0
Monthly	74	20.9
Biannually	25	7.0
Annually	15	4.2
Do not know	225	63.4
Composite practice score		
Poor practice	292	82.3
Good practice	63	17.7

## Data Availability

The data used to support the findings of this study are available from the school administration upon reasonable request at sph@gambiacollege.edu.gm.

## References

[1] CDC (2022). What is breast cancer? centers for disease control and prevention. https://www.cdc.gov/cancer/breast/basic_info/what-is-breast-cancer.htm.

[2] Ayoub N. M., Al-Taani G. M., Almomani B. A. (2021). Knowledge and practice of breast cancer screening methods among female community pharmacists in Jordan: a cross-sectional study. *International Journal of Breast Cancer*.

[3] Ji P., Gong Y., Jin M.-L., Hu X., Di G.-H., Shao Z.-M. (2020). The burden and trends of breast cancer from 1990 to 2017 at the global, regional, and national levels: results from the global burden of disease study 2017. *Frontiers in Oncology*.

[4] Seepersaud H. (2020). *Breast Cancer Knowledge , Attitude , and Screening Practices among Hispanic*.

[5] Sanyang O., Lopez-Verdugo F., Mali M. (2021). Geospatial analysis and impact of targeted development of breast cancer care in The Gambia: a cross-sectional study. *BMC Health Services Research*.

[6] Globocan (2021). The republic if the gambia fact sheets. https://gco.iarc.fr/today/data/factsheets/populations/270-the-republic-of-the-gambia-fact-sheets.pdf.

[7] WHO (2023). WHO launches new roadmap on breast cancer. https://www.who.int/news/item/03-02-2023-who-launches-new-roadmap-on-breast-cancer.

[8] Abo Al-Shiekh S. S., Ibrahim M. A., Alajerami Y. S. (2021). Breast cancer knowledge and practice of breast self-examination among female university students, Gaza. *The Scientific World Journal*.

[9] Obikunle A. F. (2016). *Barriers to Breast Cancer Prevention and Screening among African American Women*.

[10] CcAsuming-bediako A. (2018). *University of Ghana*.

[11] Kohler R. E., Gopal S., Miller A. R. (2017). A framework for improving early detection of breast cancer in sub-Saharan Africa: a qualitative study of help-seeking behaviors among Malawian women. *Patient Education and Counseling*.

[12] Pace L. E., Shulman L. N. (2016). Breast Cancer in Sub-Saharan Africa: Challenges and Opportunities to Reduce Mortality. *The Oncologist*.

[13] UTG (2023). *University of the Gambia–Knowledge, Truth & Development*.

[14] Baldeh A., Isara A. R. (2019). Knowledge of Sexually Transmitted Infections amongst Pregnant Women Attending Antenatal Clinics in West Coast Region of The Gambia. *African Journal of Reproductive Health*.

[15] Ramathuba D. U., Ratshirumbi C. T., Mashamba T. M. (2015). Knowledge, attitudes and practices toward breast cancer screening in a rural South African community. *Curationis*.

[16] Akhtari-Zavare M., Latiff L. A., Juni M. H., Said S. M., Ismail I. Z. (2015). Knowledge of female undergraduate students on breast cancer and breast self-examination in Klang Valley, Malaysia. *Asian Pacific Journal of Cancer Prevention*.

[17] Alsaraireh A., Darawad M. W. (2018). Breast cancer awareness, attitude and practices among female university students: a descriptive study from Jordan. *Health Care for Women International*.

[18] Nde F. P., Assob J. C. N., Kwenti T. E., Njunda A. L., Tainenbe T. R. G. (2015). Knowledge, attitude and practice of breast self-examination among female undergraduate students in the University of Buea. *BMC Research Notes*.

[19] Ramya Ahmad S., Asmaa Ahmad A., Nesreen Abdullah A. (2019). Awareness Level, Knowledge and Attitude towards Breast Cancer between Medical and Non-Medical University Students in Makkah Region: A Cross Sectional Study. *International Journal of Cancer and Clinical Research*.

[20] Rafique S., Waseem Z., Sheerin F. (2018). Breast Cancer Awareness, Attitude and Screening Practices Among University Students: Intervention Needed. *Biomedical Journal of Scientific & Technical Research*.

[21] Godfrey K., Agatha T., Nankumbi J. (2016). Breast cancer knowledge and breast self-examination practices among female university students in Kampala, Uganda: a descriptive study. *Oman Medical Journal*.

[22] Sama C.-B., Dzekem B., Kehbila J. (2017). Awareness of breast cancer and breast self-examination among female undergraduate students in a higher teachers training college in Cameroon. *Pan African Medical Journal*.

[23] GBoS (2020). *The Gambia Bureau of Statistics (GBoS), Ministry of Health (MoH) [The Gambia], and ICF. 2020. The Gambia Demographic and Health Survey 2019-20: Key Indicators Report*.

[24] Al-Sharbatti S. S., Shaikh R. B., Mathew E., Al-Biate M. A. S. (2013). Breast self examination practice and breast cancer risk perception among female university students in Ajman. *Asian Pacific Journal of Cancer Prevention*.

[25] Cumber S. N., Nchanji K. N., Tsoka-Gwegweni J. M. (2017). Breast cancer among women in sub-Saharan Africa: prevalence and a situational analysis. *Southern African Journal of Gynaecological Oncology*.

[26] Shimakawa Y., Bah E., Wild C. P., Hall A. J. (2013). Evaluation of data quality at the Gambia national cancer registry. *International Journal of Cancer*.

[27] Sighoko D., Bah E., Haukka J. (2010). Population-based breast (female) and cervix cancer rates in the Gambia: evidence of ethnicity-related variations. *International Journal of Cancer*.

